# ROS‐Responsive Nanobubbles for Dual‐Enhanced Ultrasound and Magnetic Resonance Imaging of Tumor Oxidative Stress

**DOI:** 10.1002/advs.202600071

**Published:** 2026-05-13

**Authors:** Wonsik Jung, Youngju Son, Dong Yun Lee, Youngeun Jeon, Muhammad Asaddudin, Sung‐Hong Park, Sangyong Jon

**Affiliations:** ^1^ Department of Biological Sciences KAIST Institute for BioCentury Korea Advanced Institute of Science and Technology (KAIST) Daejeon Republic of Korea; ^2^ Center for Precision Bio‐Nanomedicine Korea Advanced Institute of Science and Technology (KAIST) Daejeon Republic of Korea; ^3^ Department of Nuclear Medicine Asan Medical Center University of Ulsan College of Medicine Seoul Republic of Korea; ^4^ Department of Bio and Brain Engineering Korea Advanced Institute of Science and Technology (KAIST) Daejeon Republic of Korea

**Keywords:** bilirubin nanomedicine, magnetic resonance imaging, nanobubbles, oxidative stress, ultrasound imaging

## Abstract

Reactive oxygen species (ROS) are key biomarkers of oxidative stress in the tumor microenvironment (TME), yet their non‐invasive, real‐time visualization remains challenging. Here, we present biotinylated PEGylated bilirubin nanobubbles encapsulating perfluoropentane gas (bt‐PEG‐BR@PFP) as ROS‐responsive contrast agents for dual‐modality ultrasound (US) and magnetic resonance imaging (MRI). Upon ROS exposure, the bilirubin shell undergoes oxidative degradation, leading to nanobubble fusion and signal amplification in both US and T_2_*‐weighted MRI. In vitro, biotin‐mediated cellular uptake and ROS‐induced fusion were validated in A549 cancer cells. In vivo, intratumoral injection of bt‐PEG‐BR@PFP into dual‐tumor xenografts led to a >3.7‐fold increase in US signal intensity in ROS‐high A549 tumors compared to ROS‐low DU145 tumors, which was abolished by the ROS scavenger N‐acetylcysteine. Following systemic administration, the nanobubbles accumulated selectively in A549 tumors through biotin‐mediated targeting and produced ∼50‐fold higher US signal than in DU145 tumors. In contrast, the clinical agent SonoVue showed no such tumor selectivity and ROS‐responsive signal enhancement. MRI studies revealed a time‐dependent signal drop only in A549 tumors treated with bt‐PEG‐BR@PFP, consistent with ROS‐mediated nanobubble fusion. These results highlight bt‐PEG‐BR@PFP as a promising and clinically translatable platform for non‐invasive, dual‐modality imaging of tumor oxidative stress, with potential utility in various ROS‐associated pathologies.

## Graphical abstract

We report the development of biotinylated PEGylated bilirubin‐coated perfluoropentane (bt‐PEG‐BR@PFP) nanobubbles, a novel class of ROS‐activatable and dual‐modality contrast agents that amplify both ultrasound (US) and magnetic resonance imaging (MRI) signals in response to elevated ROS. In vivo studies demonstrate robust and sustained signal enhancement in both US and MRI modalities in ROS‐rich tumors.

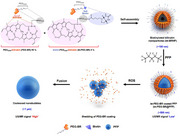

## 1 Introduction

Reactive oxygen species (ROS), including hydrogen peroxide, superoxide anion, and hydroxyl radical, are highly reactive molecules that play pivotal roles in various physiological and pathological processes [1, 2, 3]. Within the tumor microenvironment (TME), elevated ROS levels are produced not only by cancer cells but also by stromal components such as tumor‐associated macrophages and myeloid‐derived suppressor cells [4, 5]. This abnormal accumulation of ROS contributes to oxidative stress, which in turn promotes immune evasion and therapeutic resistance [4, 6, 7, 8]. Therefore, accurately detecting ROS within tumors holds great promise for assessing tumor aggressiveness and monitoring responses to chemotherapy and immunotherapy [9, 10]. While current ROS‐responsive fluorescent probes have been widely used for in vivo imaging, their clinical translation remains limited due to poor tissue penetration and suboptimal resolution in deep‐seated tumors [11, 12, 13]. These limitations underscore the pressing need for novel imaging systems capable of non‐invasive, real‐time monitoring of ROS dynamics in vivo.

Ultrasound (US) is one of the most accessible and widely used imaging modalities in clinical practice, offering a non‐invasive, cost‐effective, and real‐time approach for visualizing internal tissues. US contrast agents—particularly microbubbles—have enhanced imaging sensitivity and resolution [14, 15]. For instance, SonoVue, composed of sulfur hexafluoride (SF_6_) gas encapsulated within a phospholipid shell, has demonstrated significant improvements in ultrasound imaging resolution [16]. However, concerns regarding the greenhouse effect of SF_6_ have prompted the development of alternative agents utilizing environmentally benign gases such as perfluoropentane (PFP) [17, 18]. Despite these advances, conventional US contrast agents are incapable of providing information on pathophysiological parameters such as oxidative stress within tumors [19, 20, 21]. To address this unmet need, ROS‐responsive US contrast agents represent a promising strategy for real‐time visualization of oxidative stress in tumors.

Since magnetic resonance imaging (MRI) provides substantially higher spatial resolution and superior soft tissue contrast compared with ultrasound (US) imaging [22], a dual contrast agent that can enhance both US and MRI signals in response to elevated ROS would represent a powerful diagnostic tool for monitoring tumor progression and predicting therapeutic potential, particularly in cancers where oxidative stress is associated with drug resistance [4, 6]. Gas‐filled microbubbles used as US contrast agents, such as albumin‐coated microbubbles [22] and the clinically approved lipid‐coated SonoVue, have shown potential utility as MR contrast agents by inducing local magnetic susceptibility differences at the gas–liquid interface [22, 23, 24]. Moreover, their MR signals can be modulated by size‐dependent changes in the microbubbles [24, 25, 26]. However, none of the previously reported microbubble systems have demonstrated the capacity to simultaneously enhance US and MRI contrast in response to ROS, thereby providing functional information on oxidative stress within tumors. Alternatively, US/MRI dual contrast agents have been constructed by incorporating conventional MR probes, including iron oxide nanoparticles or Gd‐based chelates, into microbubble formulations [15]. Yet, the inherent complexity of combining distinct contrast agents and challenges in quality control during manufacturing may impede their clinical translation.

**FIGURE 1:**
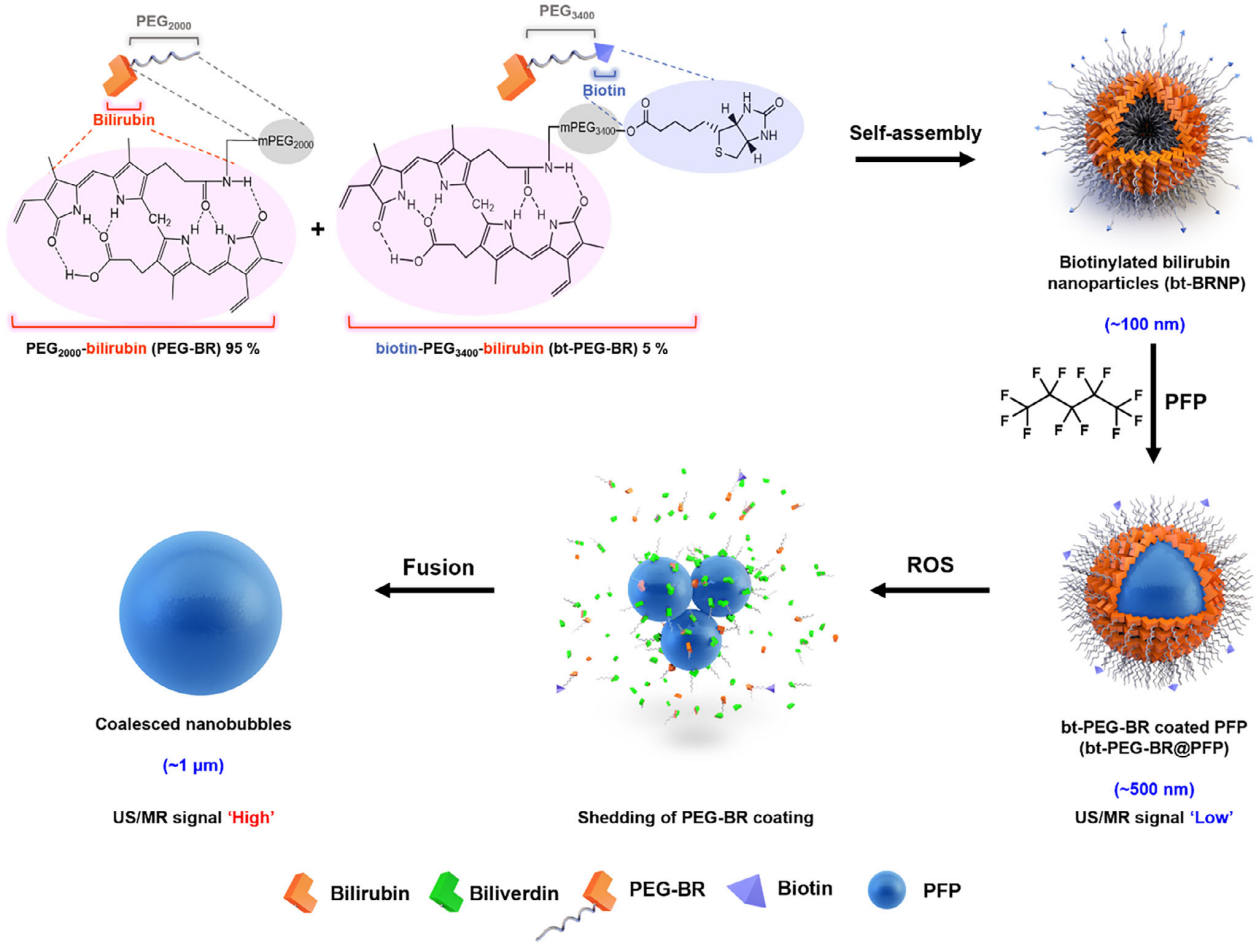
Schematic illustration for the preparation and ROS‐responsive imaging mechanism of bt‐PEG‐BR@PFP nanobubbles. bt‐PEG‐BR@PFP nanobubbles are fabricated by sonicating freshly prepared biotinylated bilirubin nanoparticles (bt‐BRNPs) with perfluoropentane (PFP) gas. Reconstitution of bt‐BRNPs (∼100 nm) with PFP gas results in a marked size increase, yielding nanobubbles of approximately 500 nm. Upon exposure to ROS, the bt‐PEG‐BR shell is shed, triggering the fusion of the exposed nanobubbles into larger microbubbles. This fusion process leads to amplified signals in both ultrasound (US) and magnetic resonance (MR) imaging modalities.

In this context, bilirubin‐based nanoparticles (BRNPs), formed via the self‐assembly of poly(ethylene glycol)‐modified bilirubin (PEG‐BR), have attracted attention for their ROS‐responsiveness and anti‐inflammatory activity [27, 28]. Upon exposure to ROS, the hydrophobic BR, within the nanoparticle core, is oxidized to the hydrophilic biliverdin (BV), leading to disassembly of the BRNPs [27, 28, 29, 30, 31, 32, 33, 34, 35, 36, 37, 38, 39]. Building upon this foundation, we developed a novel ROS‐responsive nanobubble system, comprising perfluoropentane (PFP) gas cores coated with a biotinylated PEG‐BR shell (bt‐PEG‐BR@PFP) (Figure 1). We hypothesized that ROS‐mediated oxidation of BR to BV induces a solubility transition that weakens the interfacial stability of the PEGylated shell, thereby triggering its detachment from the PFP gas core [36, 37, 38]. The exposed PFP gas cores subsequently undergo coalescence into larger bubbles, resulting in amplified US and MRI signals. This dual‐responsive nanobubble platform offers a versatile and clinically translatable tool for real‐time, non‐invasive imaging of oxidative stress in the TME.

## 2 Results and Discussion

### 2.1 Synthesis, Characterization, and ROS‐Responsiveness of Bt‐PEG‐BR@PFP Nanobubbles

**FIGURE 2:**
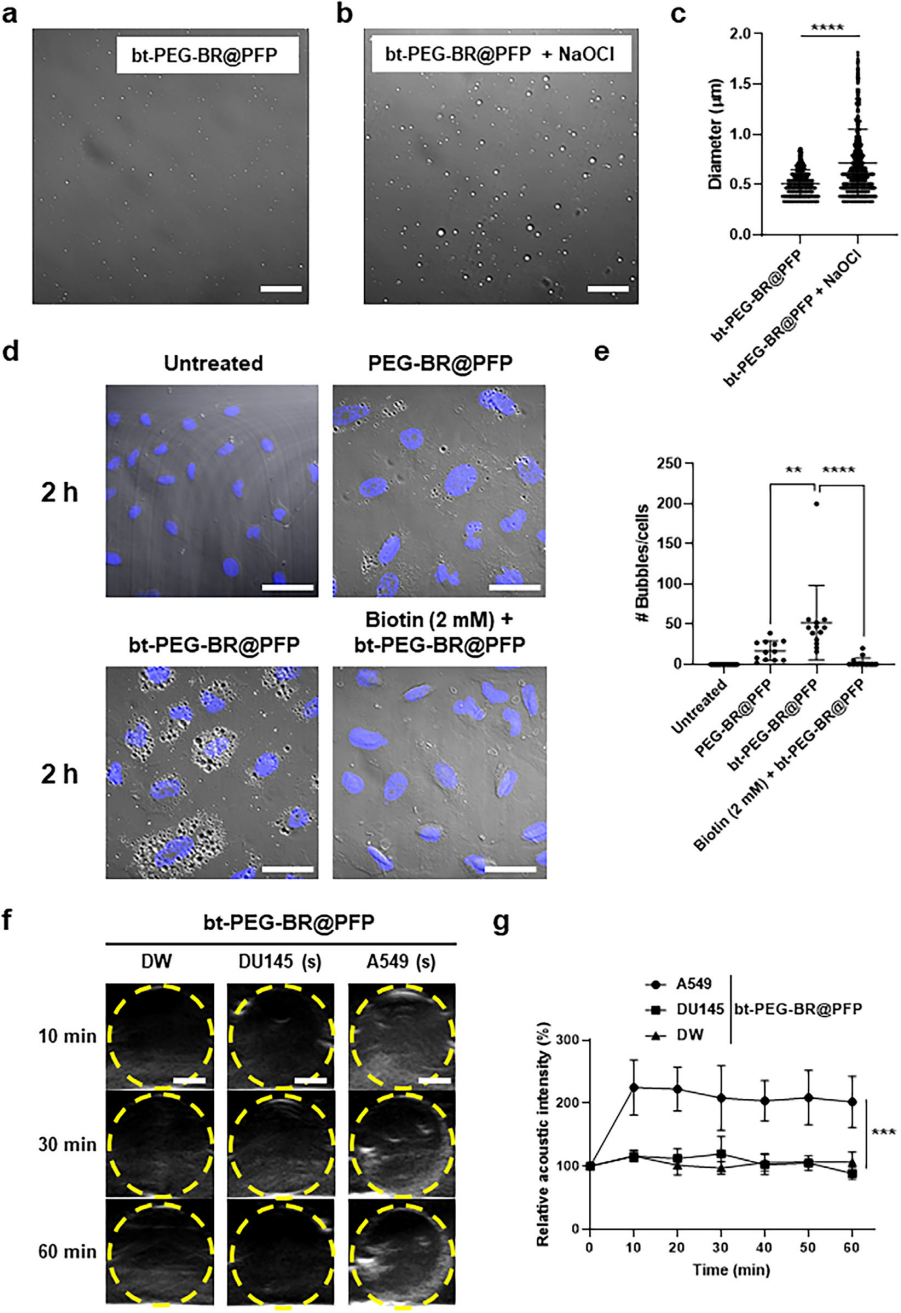
Characterization and ROS‐responsive behaviors of bt‐PEG‐BR@PFP nanobubbles. (a, b) Confocal microscopy images of bt‐PEG‐BR@PFP nanobubbles before (a) and 0.5 h after treatment with NaOCl (150 µm) (b). Scale bars, 20 µm. (c) Size distribution of bt‐PEG‐BR@PFP nanobubbles quantified from confocal microscopy images in a and b (*n* = 466 for (a) *n* = 464 for (b). Data are presented as mean ± S.D. (^****^ *p* < 0.0001, two‐tailed unpaired t‐test). (d) Bright‐field images of untreated A549 cells, A549 cells treated with PEG‐BR@PFP or bt‐PEG‐BR@PFP for 2 h, and A549 cells pretreated with biotin (2 mm) at 1 hour before bt‐PEG‐BR@PFP treatment. Numerous microbubbles are observed only in the bt‐PEG‐BR@PF‐treated cells. Scale bar, 50 µm. (e) Quantification of intracellular bubbles from the bright‐field images shown in d (*n* = 21 for untreated, *n* = 12 for PEG‐BR@PFP, *n* = 13 for bt‐PEG‐BR@PFP, and *n* = 16 for Biotin (2 mm) + bt‐PEG‐BR@PFP). Data are presented as mean ± S.D. (^**^ *p* < 0.01, ^****^ *p* < 0.0001; one‐way ANOVA). (f) Time‐lapse US phantom images of bt‐PEG‐BR@PFP nanobubbles ([PFP] = 9.69 mm) incubated with culture supernatants from ROS^high^ A549 and ROS^low^ DU145 cells (“s” denotes supernatant). Scale bar, 3 mm. (g) Quantification of relative acoustic brightness from the US images in (f). Data are presented as mean ± S.D. (*n* = 3; ^***^ *p* < 0.001, two‐tailed unpaired t‐test).

Biotinylated bilirubin nanoparticles (bt‐BRNPs) were prepared via a thin‐film hydration method using a mixture of 95 mol% poly(ethylene glycol)‐modified bilirubin (PEG‐BR) and 5 mol% biotinylated PEG‐BR (bt‐PEG‐BR) [35, 37]. Following rehydration, perfluoropentane (PFP) was added to the nanoparticle suspension, and sonication induced the formation of nanobubbles, denoted as bt‐PEG‐BR@PFP. Confocal laser scanning microscopy revealed a relatively uniform distribution of the nanobubbles with an average diameter of approximately 512.6 ± 135.4 nm (Figure 2a). Dynamic light scattering (DLS) analysis further indicated a hydrodynamic diameter of 775 ± 250 nm, which remained stable for at least 24 h, suggesting colloidal stability (Figure S1). The UV–vis absorbance spectrum of bt‐PEG‐BR@PFP was clearly distinguishable from that of bt‐BRNPs, while preserving the characteristic absorption peak of bilirubin (Figure S2).

We next evaluated the ROS responsiveness of the nanobubbles using sodium hypochlorite (NaOCl) as a model oxidant, representing superoxide anion (O_2_•^−^)‐associated ROS [40, 41, 42, 43]. Upon NaOCl exposure, confocal microscopy revealed marked enlargement of bt‐PEG‐BR@PFP nanobubbles compared with untreated controls, a change that was discernible even to the naked eye (Figure 2b). Quantitative analysis of size distribution before and after ROS treatment confirmed a significant increase in bubble diameter (Figure 2c). In agreement with these findings, DLS measurements demonstrated a significant increase in hydrodynamic diameter from ∼775 nm to ∼1,600 nm after NaOCl treatment (Figure S3a,b), suggesting ROS‐mediated coalescence of the nanobubbles. By contrast, the clinically approved US contrast agent SonoVue (SF_6_/phospholipid microbubbles) displayed no appreciable size change upon NaOCl treatment, maintaining a stable diameter of ∼1,200 nm (Figure S3c), thereby underscoring the ROS‐specific responsiveness of bt‐PEG‐BR@PFP. This ROS responsiveness was further corroborated by a clear change in the UV–vis absorption spectra and solution color, consistent with the oxidation of yellowish BR to greenish BV (Figure S4).

Given that biotin has been widely utilized as a targeting ligand for cancer cells overexpressing the biotin‐binding multivitamin transporter, we next investigated the cellular uptake of bt‐PEG‐BR@PFP nanobubbles in A549 lung carcinoma cells, which are known to overexpress the biotin transporter and produce high levels of ROS [37]. After 2 hours of incubation, a substantial number of enlarged intracellular microbubbles were observed in cells treated with bt‐PEG‐BR@PFP nanobubbles. In contrast, only a few microbubbles were detected in cells treated with PEG‐BR@PFP nanobubbles lacking the biotin ligand (Figure 2d). Moreover, pretreatment of A549 cells with an excess of free biotin (2 mm, 1 h) significantly inhibited the uptake of bt‐PEG‐BR@PFP nanobubbles (Figure S5). These findings strongly indicate that the cellular internalization of bt‐PEG‐BR@PFP nanobubbles occurs via biotin‐mediated targeting, followed by intracellular fusion of nanobubbles into microbubbles.

We next investigated the impact of ROS responsiveness on the ultrasound (US) echogenicity of bt‐PEG‐BR@PFP nanobubbles. Given that A549 human lung carcinoma cells produce markedly higher levels of ROS compared to DU145 human prostate carcinoma cells (∼6‐fold higher in culture medium), A549 and DU145 cells were selected as ROS^high^ and ROS^low^ cancer models, respectively [37]. Following incubation of bt‐PEG‐BR@PFP nanobubbles with culture media derived from either A549 or DU145 cells, dynamic US phantom imaging was performed in an agarose gel over time (Figure 2e). As early as 10 minutes post‐incubation, nanobubbles exposed to A549‐conditioned medium generated strong US signals that rapidly plateaued. In contrast, nanobubbles incubated with DU145‐conditioned medium showed negligible signal enhancement even after 60 minutes (Figure 2f). These results suggest ROS‐triggered fusion of nanobubbles in the A549 medium, leading to enhanced US signal intensity. To further validate ROS responsiveness, we tested bt‐PEG‐BR@PFP nanobubbles against two physiologically relevant ROS species: NaOCl and hydrogen peroxide (H_2_O_2_) [44, 45, 46]. The nanobubbles demonstrated concentration‐ and time‐dependent increases in US signal intensity, with a stronger response to NaOCl than to H_2_O_2_ (Figure S6). Notably, in comparison to the clinically approved SonoVue contrast agent, which has a short echo half‐life of approximately 1 min [47], bt‐PEG‐BR@PFP nanobubbles exhibited significantly prolonged echo persistence. Collectively, these findings indicate that bt‐PEG‐BR@PFP nanobubbles respond to elevated ROS levels by shedding their bt‐PEG‐BR shell, promoting fusion into larger microbubbles and thereby enhancing acoustic signal intensity during US imaging.

### 2.2 Ultrasound Imaging of Tumor ROS Using Bt‐PEG‐BR@PFP Nanobubbles after Intratumoral Injection

**FIGURE 3:**
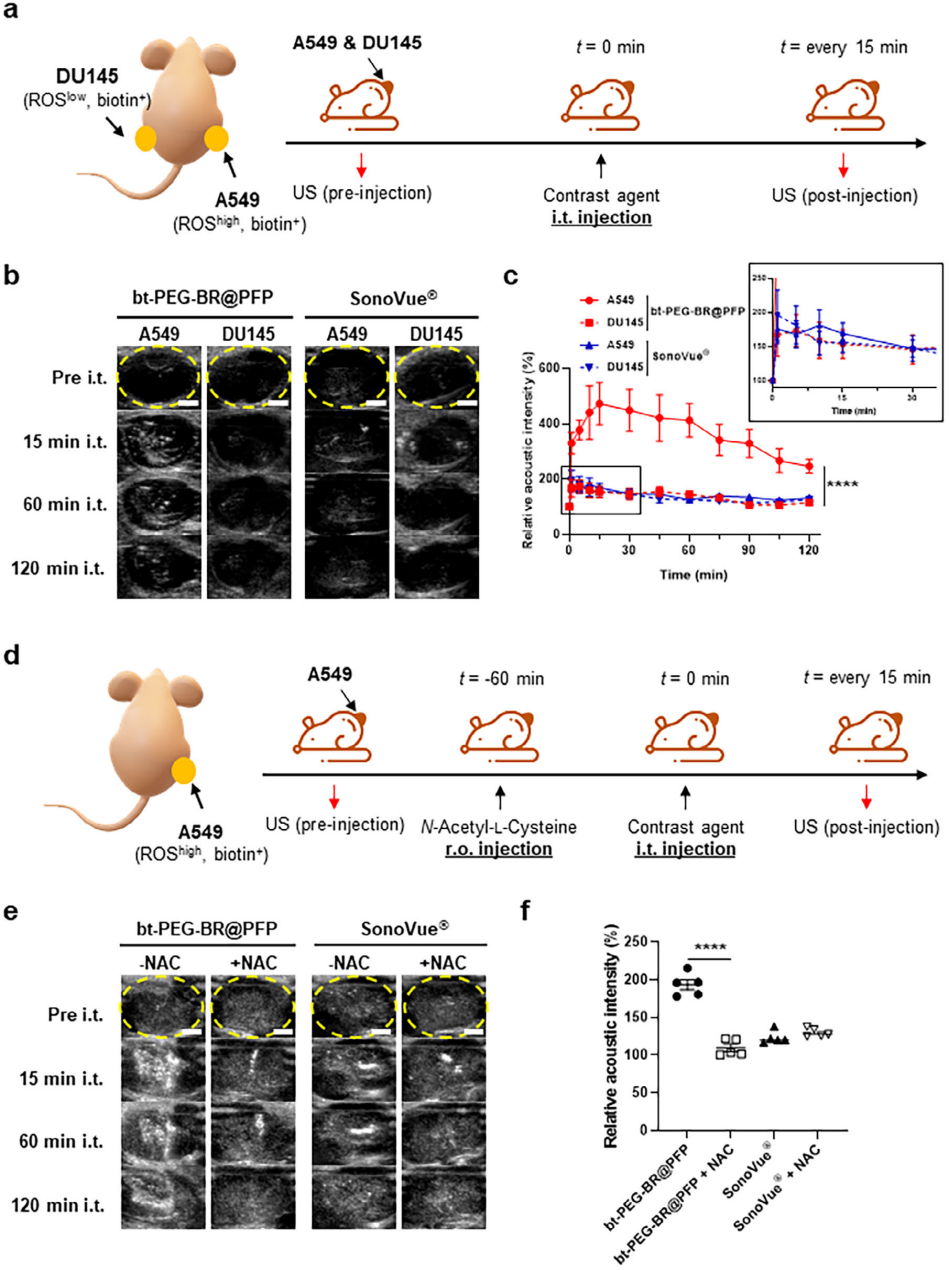
Ultrasound imaging of tumor oxidative stress following intratumoral injection of bt‐PEG‐BR@PFP nanobubbles. (a) Schematic and experimental timeline for US imaging of tumor oxidative stress using bt‐PEG‐BR@PFP or SonoVue. (b) Representative time‐dependent US images of ROS^high^ A549 and ROS^low^ DU145 tumors in mice (*n* = 5 per group) following intratumoral (i.t.) injection of bt‐PEG‐BR@PFP or SonoVue. Yellow dotted lines demarcate tumor regions. Scale bar, 2 mm. (c) Quantification of relative acoustic intensity in the tumor regions shown in (b). The inset highlights changes within the first 30 minutes post‐injection. Data are presented as mean ± S.E.M. (^****^ *p* < 0.0001, two‐tailed unpaired t‐test). (d) Schematic and schedule for N‐acetylcysteine (NAC) pretreatment experiments in A549 tumor‐bearing mice. (e) Representative time‐dependent US images of ROS^high^ A549 tumors with or without NAC pretreatment (intravenous; 125 mg/kg, 1 h prior), followed by i.t. injection of bt‐PEG‐BR@PFP or SonoVue (*n* = 5 per group). Tumor regions are outlined with yellow dotted lines. Scale bar, 2 mm. (f) Quantification of relative acoustic intensity from the tumor regions shown in (e). Data are presented as mean ± S.E.M. (*n* = 5; ^****^ *p* < 0.0001, two‐tailed unpaired t‐test).

To evaluate the in vivo capability of bt‐PEG‐BR@PFP nanobubbles for ROS imaging, we employed a dual‐tumor xenograft model in Balb/c nude mice, with ROS^high^ A549 lung cancer and ROS^low^ DU145 prostate cancer cells implanted on opposite flanks (Figure 3a). Dihydroethidium (DHE) staining confirmed significantly higher ROS levels in A549 tumors compared to DU145 tumors, consistent with previous reports (Figure S7). Following intratumoral (i.t.) injection of bt‐PEG‐BR@PFP nanobubbles into both tumors, real‐time ultrasound (US) images were acquired to monitor signal changes over time (Figure 3a). In ROS^high^ A549 tumors, US brightness markedly increased, reaching a peak at 15 min post‐injection, followed by a gradual decline. In contrast, ROS^low^ DU145 tumors exhibited only a slight signal enhancement (Figure 3b). Quantitative analysis revealed a maximum US signal intensity increase of approximately 373% in A549 tumors at 15 min post‐injection, relative to baseline (Figure 3c). This robust signal amplification was not observed in DU145 tumors. As a control, tumors injected with the clinical US contrast agent SonoVue displayed only modest and comparable signal increases in both tumor types, with no ROS‐dependent enhancement (Figure 3b,c). These results support the unique ROS‐responsiveness of bt‐PEG‐BR@PFP nanobubbles, enabling selective visualization of oxidative stress within tumors via ultrasound imaging.

To further confirm that the US signal enhancement was mediated by intratumoral ROS, we pretreated A549 tumor‐bearing mice with N‐acetyl‐L‐cysteine (NAC), a widely used ROS scavenger [48, 49]. NAC was administered via retro‐orbital injection one hour prior to i.t. injection of bt‐PEG‐BR@PFP nanobubbles (Figure 3d). Pretreatment with NAC substantially suppressed US signal enhancement in the tumor region, as compared to non‐pretreated controls (Figure 3e,f). In contrast, SonoVue‐injected tumors exhibited no change in signal intensity regardless of NAC administration. These findings collectively demonstrate that bt‐PEG‐BR@PFP nanobubbles serve as a sensitive US contrast agent for detecting ROS levels in vivo. Their responsiveness to ROS—confirmed by both dual‐tumor models and pharmacological scavenging—highlights their potential utility for real‐time, non‐invasive imaging of tumor oxidative stress.

### 2.3 Ultrasound Imaging of ROS in Tumor‐Bearing Mice Following Systemic Administration of bt‐PEG‐BR@PFP Nanobubbles

**FIGURE 4:**
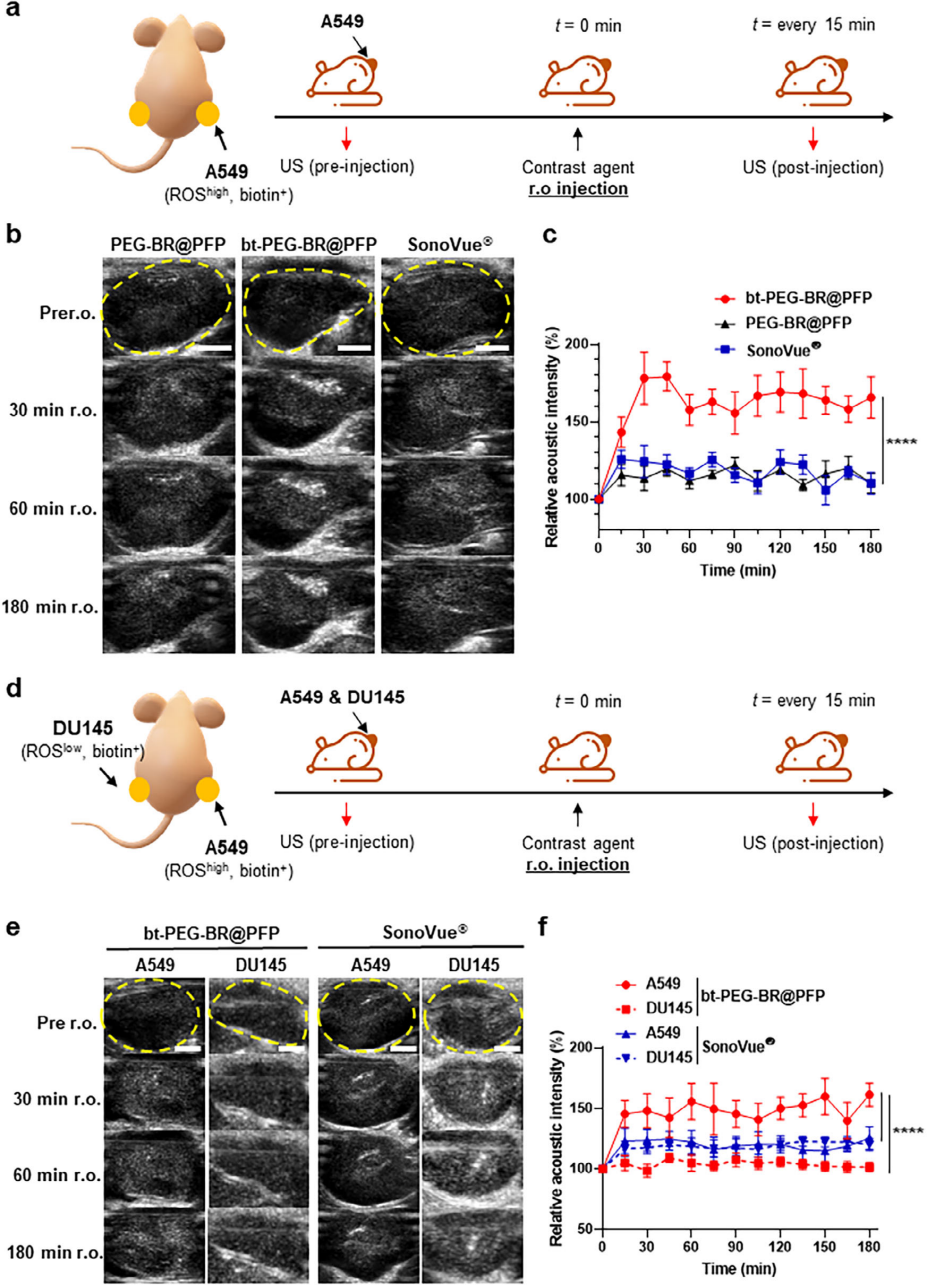
Ultrasound imaging of tumor oxidative stress following systemic administration of bt‐PEG‐BR@PFP nanobubbles. (a) Schematic and timeline for US imaging experiments comparing bt‐PEG‐BR@PFP, PEG‐BR@PFP, and SonoVue following retro‐orbital (r.o.) injection. (b) Representative time‐dependent US images of tumors in ROS^high^ A549 tumor‐bearing mice after r.o. injection of bt‐PEG‐BR@PFP (*n* = 7), PEG‐BR@PFP (*n* = 5), or SonoVue (*n* = 5). Tumor regions are outlined by yellow dotted lines. Scale bar, 2 mm. (c) Quantitative analysis of relative acoustic intensity in the tumor regions shown in (b) comparing bt‐PEG‐BR@PFP (red line), PEG‐BR@PFP (black line), and SonoVue (blue line). Data are presented as mean ± S.E.M. (^****^ *p* < 0.0001, two‐tailed unpaired t‐test). (d) Schematic and timeline for additional US imaging comparing bt‐PEG‐BR@PFP and SonoVue following r.o. injection in different tumor models. (e) Representative time‐dependent US images of ROS^high^ A549 and ROS^low^ DU145 tumors in mice following r.o. injection of bt‐PEG‐BR@PFP or SonoVue (*n* = 5 per group). Yellow dotted lines mark tumor boundaries. Scale bar, 2 mm. (f) Quantification of relative acoustic intensity in the tumor regions shown in (e). Data are presented as mean ± S.E.M. (*n* = 5; ^****^ *p* < 0.0001, two‐tailed unpaired t‐test).

Given that systemic administration is more clinically relevant than intratumoral (i.t.) injection, we next assessed the imaging performance of bt‐PEG‐BR@PFP nanobubbles following retro‐orbital (r.o.) injection in Balb/c nude mice bearing ROS^high^ A549 lung tumors, which overexpress the biotin‐binding multivitamin transporter (Figure 4a). As controls, we used PEG‐BR@PFP nanobubbles lacking the biotin ligand and the clinically approved SonoVue microbubbles. Although the size of bt‐PEG‐BR@PFP nanobubbles (∼500 nm) is larger than the range typically considered optimal for tumor targeting via the enhanced permeability and retention (EPR) effect, actual EPR‐mediated accumulation is influenced by multiple factors, including particle size, shape, and mechanical properties. In this context, we hypothesized that the intrinsic softness of bt‐PEG‐BR@PFP nanobubbles may facilitate their extravasation and penetration into tumor tissues more effectively than rigid nanoparticles of comparable size [50]. US imaging was performed at 15‐min intervals post‐injection. Mice treated with bt‐PEG‐BR@PFP nanobubbles exhibited a progressive increase in acoustic intensity at the tumor site, reaching a 3.3‐fold higher signal enhancement than the PEG‐BR@PFP group at 30 minutes post‐injection (Figure 4b,c). This significant enhancement is attributed to both biotin‐mediated active targeting and ROS‐triggered nanobubble fusion within the tumor microenvironment. In contrast, SonoVue—which lacks both tumor‐targeting ability and ROS responsiveness—showed only marginal and stable signal enhancement for up to 3 hours.

To dissect the individual contributions of biotin targeting and ROS sensitivity, we employed a dual‐tumor model in which ROS^high^ A549 and ROS^low^ DU145 tumors, both of which express the biotin multivitamin transporter, were established on opposite flanks of the same mouse. Mice were systemically administered bt‐PEG‐BR@PFP nanobubbles or SonoVue via r.o. injection (Figure 4d). Strikingly, US imaging revealed intense and time‐dependent signal enhancement in A549 tumors, while DU145 tumors showed minimal changes (Figure 4e). Quantitative analysis demonstrated that bt‐PEG‐BR@PFP nanobubbles generated a ∼50‐fold higher signal in A549 tumors compared to DU145 tumors (Figure 4f). The significant disparity in US signal between the two tumor types—despite comparable expression levels of the biotin transporter—highlights the critical role of ROS in mediating nanobubble fusion and subsequent acoustic signal amplification. As expected, SonoVue showed no significant difference in signal intensity between the two tumor models, further confirming the ROS‐specific responsiveness of bt‐PEG‐BR@PFP. Together, these findings demonstrate that bt‐PEG‐BR@PFP nanobubbles can effectively localize to tumors via biotin‐mediated active targeting and generate robust ultrasound signal enhancement through ROS‐triggered nanobubble fusion. This dual functionality offers a powerful and non‐invasive strategy for detecting oxidative stress in tumors, supporting the potential of bt‐PEG‐BR@PFP as a clinically translatable ROS‐responsive US contrast agent.

### 2.4 MRI Imaging of Tumor ROS Using Bt‐PEG‐BR@PFP Nanobubbles after Systemic Injection

**FIGURE 5:**
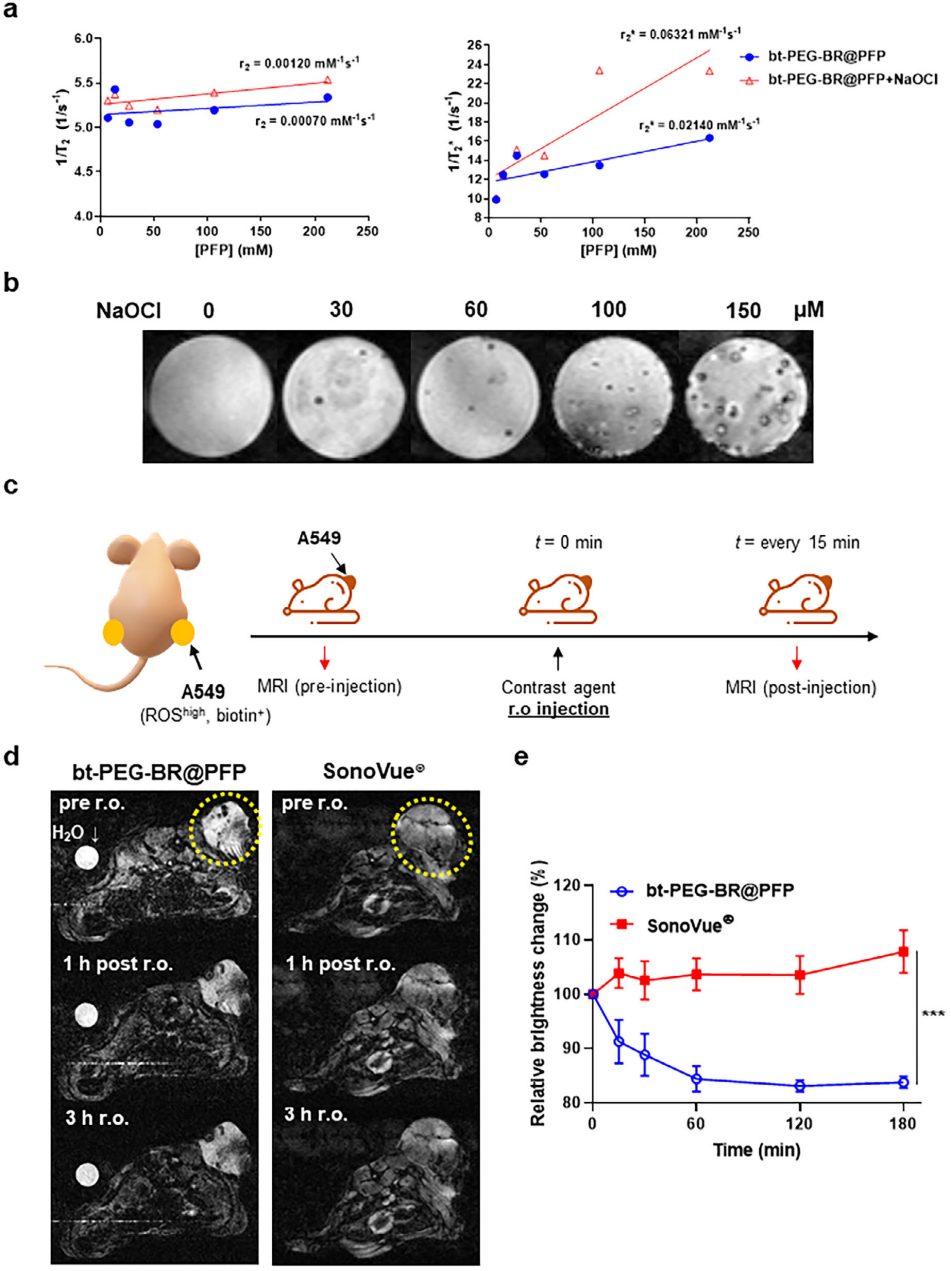
T_2_*‐weighted magnetic resonance imaging of tumor oxidative stress following systemic administration of bt‐PEG‐BR@PFP nanobubbles. (a) T_2_ relaxation rate (r_2_, Left) and T_2_* relaxation rate (r_2_*, Right) of bt‐PEG‐BR@PFP nanobubbles in the absence or presence of NaOCl (150 µm). (b) T_2_*‐weighted MR phantom images of bt‐PEG‐BR@PFP nanobubbles ([PFP] = 9.69 mm) incubated with increasing concentrations of NaOCl for 30 minutes. Each tube was independently prepared prior to scanning. (c) Schematic and experimental timeline for in vivo MRI imaging of tumor oxidative stress using bt‐PEG‐BR@PFP or SonoVue. (d) Representative time‐dependent transverse T_2_*‐weighted MR images of ROS^high^ A549 tumors following retro‐orbital (r.o.) injection of bt‐PEG‐BR@PFP or SonoVue (*n* = 3 per group). Yellow dotted lines delineate tumor boundaries. (e) Quantification of changes in relative signal intensity (darkness) in the tumor regions shown in (c), comparing bt‐PEG‐BR@PFP (blue line) and SonoVue (red line). Data are presented as mean ± S.E.M. (^***^ *p* < 0.001, two‐tailed unpaired t‐test). All MR images were acquired using a 3T MRI scanner.

Given that gas‐based US contrast agents have shown potential for dual US/MRI imaging, we next assessed the feasibility of employing bt‐PEG‐BR@PFP nanobubbles as MRI contrast agents. We first measured the concentration‐dependent T_2_, T_2_*, and magnetic susceptibility of the nanobubbles in the absence or presence of NaOCl. Increasing nanobubble concentrations caused only a minor reduction in T_2_ values (<10%) but led to a more pronounced decrease in T_2_* values (Figure S8), consistent with the increased magnetic susceptibility (Figure S9). It has been shown that gas‐filled particles in aqueous media induce substantial local magnetic susceptibility differences at the gas–liquid interface, thereby producing magnetic field distortions that accelerate the dephasing of nearby water proton spins and markedly shorten the T_2_* relaxation time, enhancing MR signal sensitivity [10, 11]. As expected, r_2_* relaxivity measurements revealed that NaOCl‐triggered enlargement of bt‐PEG‐BR@PFP nanobubbles resulted in approximately threefold higher r_2_* relaxivity compared with untreated bt‐PEG‐BR@PFP nanobubbles, consistent with the increased magnetic susceptibility (from 0.60 ppm/m to 1.04 ppm/M after NaOCl triggering) (Figure S9), while r_2_ relaxivity showed only minimal change (Figure 5a).

Based on this T_2_* shortening effect, we acquired T_2_*‐weighted MRI phantom images for bt‐PEG‐BR@PFP nanobubbles treated with increasing concentrations of NaOCl to simulate ROS exposure (Figure 5b; Figure S10). As the NaOCl concentration increased, the number and intensity of dark spots in the images also increased, indicating progressive fusion of nanobubbles into larger microbubbles. These fused structures, which are more readily detectable by MRI due to increased magnetic susceptibility effects, also became visually apparent, further confirming ROS‐responsiveness. This enhanced detectability can be attributed to the coalescence of individual nanobubbles into larger microbubbles within the tumor microenvironment.

To investigate in vivo MRI contrast enhancement, T_2_*‐weighted coronal MRI images were obtained over time following retro‐orbital (r.o.) injection of either bt‐PEG‐BR@PFP nanobubbles or SonoVue in A549 lung tumor‐bearing mice, which overexpress the biotin multivitamin transporter and exhibit high levels of ROS (Figure 5c). In mice treated with SonoVue, no significant changes in MRI signal intensity were observed at the tumor site up to 3 h post‐injection. In contrast, bt‐PEG‐BR@PFP‐treated mice exhibited a time‐dependent decrease in signal intensity at the tumor regions, indicating enhanced T_2_* contrast. This effect is consistent with active tumor accumulation via biotin‐mediated targeting, followed by ROS‐triggered nanobubble fusion (Figure 5d,e). Quantitative analysis revealed a pronounced and sustained signal drop in the bt‐PEG‐BR@PFP group, in stark contrast to the negligible signal change observed in the SonoVue group. These data clearly demonstrate that the bt‐PEG‐BR@PFP nanobubbles serve as an effective ROS‐responsive MRI contrast agent in addition to their established US functionality. Taken together, bt‐PEG‐BR@PFP nanobubbles exhibit dual‐modal contrast enhancement capabilities for both US and MRI imaging. Their tumor‐specific accumulation and ROS‐induced fusion result in amplified signal responses, offering a robust platform for non‐invasive detection of tumor oxidative stress.

### 2.5 Biodistribution, Pharmacokinetics, and Toxicity of Bt‐PEG‐BR@PFP Nanobubbles

To evaluate the biodistribution and pharmacokinetic behavior of bt‐PEG‐BR@PFP nanobubbles, cypate‐loaded nanobubbles were prepared (Figure S11a). Cypate, a lipophilic near‐infrared cyanine dye, was successfully incorporated into the nanobubble shell. Following systemic administration (r.o.) of cypate‐loaded bt‐PEG‐BR@PFP nanobubbles (1.4 mg cypate/kg) into A549 tumor‐bearing mice, whole‐body fluorescence imaging was performed at predetermined time points using an in vivo imaging system (IVIS). Progressive accumulation of fluorescence signals around tumor sites was observed over time and remained detectable up to 6 h post‐injection (Figure S11b). Ex vivo IVIS imaging of major organs harvested at 6 h revealed a predominant fluorescence signal in the liver, consistent with the typical hepatobiliary clearance of nanoscale materials, with relatively strong signals also detected in the kidneys (Figure S11c). Notably, excised tumors from mice receiving cypate‐loaded bt‐PEG‐BR@PFP nanobubbles exhibited significantly higher fluorescence intensity than those from mice receiving free cypate (Figure S11d,e), indicating enhanced tumor accumulation mediated by the nanobubble platform.

A pharmacokinetic study was conducted in normal ICR mice following systemic injection (r.o.) of cypate‐loaded bt‐PEG‐BR@PFP nanobubbles (5 mg cypate/kg). Blood samples were collected at 0 min, 5 min, and 30 min and 1 h, 2 h, 4 h, 8 h, 12 h, 24 h, and 48 h post‐injection, and cypate concentration was quantified by fluorescence. The blood concentration‐time profile was fitted to a two‐compartment model (Figure S12), showing a biphasic decline with a rapid distribution (initial circulation) phase (t_1/2_α = 0.446 h), followed by a slower terminal phase (t_1/2_β = 5.29 h), with a systemic exposure AUC_0‐inf_ of 531.59 µg/mL*h. Although the initial blood circulation half‐life is not markedly prolonged, tumor accumulation of bt‐PEG‐BR@PFP nanobubbles was already evident at 15 min after injection (Figure 4), indicating that the observed circulation kinetics are sufficient to support effective tumor imaging.

Although the safety of both PEGylated bilirubin nanoparticles and PFP‐based US contrast agents has been supported in prior clinical evaluations [51, 52, 53, 54, 55], we further assessed the in vivo toxicity of bt‐PEG‐BR@PFP nanobubbles in normal ICR mice. Following systemic administration (r.o.) (100 mg PFP/kg), mice were monitored for 7 days. No significant differences in body weight or general health status were observed between nanobubble‐treated and PBS‐treated groups (Figure S13a). Hematological parameters measured on day 7 showed no statistically significant alterations (Figure S13b). In addition, serum biochemical markers of hepatic and renal function remained within normal physiological ranges, indicating the absence of systemic toxicity. Histopathological examination of major organs—including heart, lung, liver, spleen, and kidney—revealed no evidence of tissue damage, inflammatory infiltration, or structural abnormalities in nanobubble‐treated mice compared with PBS‐treated controls (Figure S13c).

Collectively, these findings demonstrate that bt‐PEG‐BR@PFP nanobubbles exhibit favorable biodistribution, adequate circulation time, and little in vivo toxicity, supporting their translational potential as a safe and effective US/MRI imaging platform for tumor oxidative stress. Although the present system is designed as an imaging probe intended for single‐dose administration, comprehensive evaluation of long‐term and repeated‐dose toxicity will be necessary for future clinical translation.

## 3 Conclusions

In this study, we developed a novel class of ROS‐responsive nanobubbles, bt‐PEG‐BR@PFP, engineered to enable enhanced dual‐modality imaging of tumor oxidative stress. These nanobubbles are designed to exploit the oxidative detachment of the bilirubin shell to trigger fusion of the PFP gas core, thereby amplifying signal intensity detectable by both US and MRI. In vivo studies with both systemic and intratumoral administration demonstrated selective accumulation and activation of bt‐PEG‐BR@PFP in ROS‐rich tumor microenvironments, facilitated by biotin‐mediated targeting and intrinsic redox sensitivity. Compared with the clinically approved agent SonoVue, bt‐PEG‐BR@PFP nanobubbles achieved superior and more sustained imaging contrast, underscoring their promise as a next‐generation diagnostic platform. Nonetheless, their clinical applicability may be limited to specific settings, as US is widely accessible but resolution‐restricted, whereas MRI provides superior resolution yet is constrained by limited availability, higher cost, and longer acquisition times. Collectively, these findings establish bt‐PEG‐BR@PFP nanobubbles as a promising tool for non‐invasive, real‐time visualization of oxidative stress within the tumor microenvironment, with broad translational potential in oncology, inflammatory disease, and redox biology.

## 4 Materials and Methods

### 4.1 Materials

Bilirubin (BR) was purchased from Tokyo Chemical Industry Co., Ltd. (Tokyo, Japan). Carbonyldiimidazole, sodium hypochlorite (NaOCl), N‐acetyl‐L‐cysteine (NAC), and sodium carbonate were obtained from Sigma‐Aldrich (St. Louis, MO, USA). Methoxy poly(ethylene glycol)‐amine (mPEG_2000_‐NH_2_) and biotin‐PEG_3400_‐NH_2_ were purchased from FutureChem (Seoul, Korea). Perfluoropentane (PFP) was obtained from Oakwood Products, Inc. (Estill, SC, USA). The Amersham ECL Prime detection kit was purchased from GE Healthcare (Chicago, IL, USA). SonoVue was purchased from Bracco Imaging (Milan, Italy).

### 4.2 Cell Lines

The human lung carcinoma cell line A549 (RRID: CVCL_0023) and the human prostate carcinoma cell line DU145 (RRID: CVCL_0105) were obtained from the American Type Culture Collection (ATCC, Manassas, VA, USA). Cells were routinely tested and confirmed to be free of mycoplasma contamination. A549 cells were maintained in Ham's F‐12K medium supplemented with 10% fetal bovine serum (FBS; Welgene Inc., Gyeongsan, Korea), and DU145 cells were cultured in Dulbecco's Modified Eagle Medium (DMEM) supplemented with 10% FBS. All cells were incubated at 37°C in a humidified atmosphere containing 5% CO_2_.

### 4.3 Animals

Female ICR mice (7 weeks old, 26–28 g) and BALB/c nude mice (6 weeks old, 18–20 g) were purchased from Orient Bio, Inc. (Seongnam, Korea). Animals were housed under pathogen‐free conditions at the Korea Advanced Institute of Science and Technology (KAIST) and Asan Medical Center, maintained at 24°C with 50% relative humidity. All animal experiments were conducted under isoflurane anesthesia and approved by the Institutional Animal Care and Use Committees of KAIST (KA2022‐011) and Asan Medical Center (2022‐14‐252), in accordance with institutional and national guidelines.

### 4.4 Preparation of PEG‐BR@PFP and bt‐PEG‐BR@PFP Nanobubbles

PEG_2000_‐BR and biotin‐PEG_3400_‐BR (bt‐PEG‐BR) were synthesized as described previously [27, 35, 37]. To prepare bilirubin nanoparticles (BRNPs), solutions of PEG_2000_‐BR (0.6 mg, 0.23 µmol) and bt‐PEG_3400_‐BR (0–0.02 µmol) in 0.1 mL of chloroform were evaporated using a rotary pump to form thin films. The films were rehydrated with 950 µL of distilled water, followed by the addition of 50 µL PFP, and subjected to sonication (bath sonicator, 1 min) to generate PEG‐BR@PFP or bt‐PEG‐BR@PFP nanobubbles.

### 4.5 Characterization of Bt‐PEG‐BR@PFP Nanobubbles

Hydrodynamic diameter and ζ‐potential were measured using dynamic light scattering (Zetasizer Nano ZS90, Malvern Instruments, UK). Nanobubble morphology and size distribution were assessed using confocal laser scanning microscopy (LSM880, Carl Zeiss, Germany), with analysis performed using ZEN 3.0 software. UV–vis absorption spectra were recorded using a Spark 10 m multimode microplate reader (Tecan, Zürich, Switzerland).

### 4.6 Confocal Cellular Imaging

To visualize cellular uptake, A549 cells were treated with either PEG‐BR@PFP or bt‐PEG‐BR@PFP nanobubbles for 2 h and imaged using confocal laser scanning microscopy (LSM880, Carl Zeiss, Germany). For receptor blocking experiments, a separate group of A549 cells was pre‐incubated with 2 mm biotin for 1 h prior to the addition of bt‐PEG‐BR@PFP nanobubbles. The number of intracellular nanobubbles was quantified using ZEN 3.0 software.

### 4.7 In Vitro ROS Quantification

A549 and DU145 cells were seeded in 6‐well plates at 1 × 10^5^ cells/well and incubated for 40 h in their respective growth media. The supernatants were collected post‐centrifugation and analyzed for extracellular ROS levels using a chemiluminescence‐based assay (Amersham ECL Prime, GE Healthcare) according to the manufacturer's instructions.

### 4.8 Ex Vivo ROS Detection in Tumor Tissues

Subcutaneous xenograft tumors were established by inoculating 100 µL of A549 or DU145 cell suspensions (2 × 10^6^ cells/mL) into the left and right flanks of BALB/c nude mice, respectively. When tumors reached ∼250 mm^3^, they were excised, fixed in 4% neutral‐buffered formalin, and stained with 500 µM of dihydroethidium (DHE) for 30 min at 37°C to detect superoxide production. ROS distribution was visualized by confocal laser scanning microscopy (LSM880, Carl Zeiss, Germany).

### 4.9 MR Imaging and Phantom Imaging

T_2_*‐weighted MR phantom imaging of bt‐PEG‐BR@PFP nanobubbles (in distilled water) and SonoVue was performed before and after treatment with NaOCl using a 3T MRS 3000 scanner (MR Solutions, UK; bore size: 17 cm). To prevent the movement of bubbles, 0.5% agar was added to increase the viscosity of the medium within the tubes. Imaging parameters: gradient echo sequence; TR = 1100 ms; TE = 30 ms; flip angle = 60°; field of view (FOV) = 40 × 40 mm^2^; slice thickness = 1 mm; 15 slices; matrix size = 256 × 256. The experiment was done using the multi‐echo gradient echo sequence (time to repeat (TR) = 1100 ms, echo time (TE) = 6 – 120.9 ms with 6.1‐ms echo spacing (20 echoes), flip angle (FA) = 30°, field of view (FOV) = 45 × 45 mm^2^, slice thickness = 1 mm^2^ with 8 slices, and matrix size = 256 × 256) to measure T_2_* and magnetic susceptibility, and using the multi‐echo spin‐echo sequence (time to repeat (TR) = 8200 ms, echo time (TE) = 25–1000 ms with 25‐ms echo spacing (40 echoes)) to measure T_2_. T_2_* and T_2_ values were measured using least‐squares‐based curve fitting. The relaxivity, r_2_*, was calculated from the equation r_2_* = ΔR_2_*/[C], where ΔR_2_* was obtained from multiple echo times (R_2_* = 1/T_2_*) and [C] is the PFP concentration measured in mm. Magnetic susceptibility was quantitatively measured using morphology‐enabled dipole inversion [56].

### 4.10 Ultrasound Phantom Imaging

Ultrasound phantom imaging was performed by loading the samples (bt‐PEG‐BR@PFP nanobubbles or SonoVue) into a custom‐fabricated mold made of 2 wt% agar. Ultrasound imaging of nanobubbles (in DW) before and after NaOCl treatment was conducted using a Philips iU22 ultrasound system in gray‐scale mode (mechanical index = 0.6) with a 12 MHz linear transducer (L12‐5).

### 4.11 In Vivo MRI and Ultrasound Imaging

Tumors were established by subcutaneous injection of 100 µL A549 suspended in Ham's F‐12K medium or DU145 cells suspended in DMEM medium (2 × 10^6^ cells/mL) into the flanks of BALB/c nude mice. When tumor volumes reached ∼100 mm^3^, mice received intratumoral or intravenous (retro‐orbital) injections of bt‐PEG‐BR@PFP nanobubbles (100 mg PFP/kg) or SonoVue. In NAC pre‐treatment studies, mice were intravenously (r.o.) injected with NAC (125 mg/kg) 1 h before nanobubble administration. Temporal changes in tumor brightness were evaluated by acquiring a series of scans at predetermined time points. The mice's physiological condition, including respiration and temperature, was monitored for the duration of the scan. T_2_*‐weighted dynamic MR imaging was performed using a 3T scanner with the following parameters: gradient echo sequence; flip angle = 50°; TR/TE = 1100/30 ms; matrix size = 256 × 256; temporal resolution = 3.5 min; scan duration = 3 h; FOV = 40 × 40 mm^2^; 15 slices. Ultrasound imaging was conducted using gray‐scale mode (mechanical index = 0.6) and a 12 MHz linear transducer (L15‐7io). For quantitative analysis, regions of interest (ROIs) encompassing the tumor area were manually defined on the acquired images to measure the mean signal intensity.

### 4.12 Biodistribution Study

Tumor xenografts were established by subcutaneous injection of 100 µL of A549 cells suspended in Ham's F‐12K medium into the flanks of BALB/c nude mice. When the tumor volume reached an average of approximately 300 mm^3^, 100 µL of cypate‐loaded bt‐PEG‐BR@PFP nanobubbles (equivalent to 1.4 mg cypate/kg) were administered via intravenous (retro‐orbital) injection. Cypate‐loaded bt‐PEG‐BR@PFP nanobubbles were prepared by forming thin films composed of cypate (0.7 mg, 1.1 µmol), PEG‐BR (3.2 mg, 1.3 µmol), and bt‐PEG‐BR (2.5 mg, 0.065 µmol). The films were rehydrated with 250 µL of PBS and 250 µL of PFP, followed by sonication to generate nanobubbles. Free cypate was removed by gel filtration using a Sephadex G‐50 column, and only cypate‐loaded bt‐PEG‐BR@PFP nanobubbles were collected. The amount of loaded cypate was quantified using a microplate reader (Tecan, Zürich, Switzerland) at an excitation wavelength of 780 nm and an emission wavelength of 820 nm. For comparison, free cypate was prepared as a stock solution in dimethyl sulfoxide (DMSO) and subsequently diluted with PBS to achieve the same cypate dose as that loaded in the nanobubbles, with the final DMSO concentration adjusted to 5% (v/v) prior to retro‐orbital injection. At predetermined time points (1 h, 3 h, and 6 h), in vivo fluorescence images were acquired under isoflurane anesthesia using a Xenogen Lumina In Vivo Imaging System (IVIS; PerkinElmer, Waltham, MA, USA) equipped with an indocyanine green (ICG) filter channel and an exposure time of 5 s. At 6 h post‐injection, mice were euthanized, and tumors as well as major organs (heart, lung, liver, kidney, and spleen) were harvested. Ex vivo fluorescence images were obtained using the IVIS system under the same imaging conditions (ICG filter channel, exposure time = 5 s). Fluorescence intensities of tumors were quantified using the instrument's analysis software.

### 4.13 Pharmacokinetic Analysis

Cypate‐loaded bt‐PEG‐BR@PFP nanobubbles (5 mg cypate/kg) were prepared in PBS. A total volume of 100 µL was administered via systemic injection (r.o.) to 7‐week‐old female ICR mice under isoflurane anesthesia. At predetermined time points post‐injection (0 min, 5 min, 30 min, 1 h, 2 h, 4 h, 8 h, 12 h, 24 h, and 48 h), 200 µL of blood was collected via retro‐orbital sampling, and serum was obtained by centrifugation. The collected serum samples were diluted 1:1 (v/v) with DMSO and centrifuged at 10 000 × g for 5 min. The supernatants were collected, and fluorescence intensity was measured using a microplate reader (Tecan, Zürich, Switzerland) at an excitation wavelength of 780 nm and an emission wavelength of 820 nm to quantify the concentration of cypate‐loaded bt‐PEG‐BR@PFP nanobubbles (µg/mL) in the serum. Pharmacokinetic parameters were calculated using PKSolver software based on a two‐compartment model.

### 4.14 In Vivo Toxicity Study

The safety evaluation was conducted using 7‐week‐old female ICR mice. PBS or bt‐PEG‐BR@PFP nanobubbles (100 mg PFP/kg) were intravenously (r.o.) administered in a total volume of 100 µL per mouse. Body weight and clinical signs were monitored daily for 7 days following injection to assess potential systemic toxicity. At 7 days post‐administration, mice were euthanized, and blood samples were collected. Whole blood was collected into ethylenediaminetetraacetic acid (EDTA)‐coated tubes for hematological analysis (WBC, Neu, Lym, Mono, Eos, PLT, RBC, HGB, HCT, MCV, MCH, and MCHC). For serum biochemical analysis, blood samples were collected into serum‐separating tubes (SST) and centrifuged to obtain serum. The resulting serum was used for biochemical analysis (ALT, AST, ALP, Alb, T‐chol, BUN, and Crea). Major organs, including the heart, lung, spleen, liver, and kidney, were harvested and fixed in 10% neutral‐buffered formalin. The tissues were embedded in paraffin, sectioned, and stained with hematoxylin and eosin (H&E) for histopathological evaluation. All hematological, biochemical, and histopathological analyses were performed in a blinded manner. Investigators responsible for sample analysis and histological assessment were blinded to the treatment groups until completion of data analysis.

### 4.15 Statistical Analysis

The number of independent samples (n) is indicated in each figure legend. Quantitative data are expressed as mean ± standard deviation (S.D.) or mean ± standard error of the mean (S.E.M.) as indicated. Statistical significance was assessed using two‐tailed unpaired Student's t‐tests or one‐way ANOVA with GraphPad Prism 8.2.1. A p value < 0.05 was considered statistically significant. Levels of significance are denoted as follows: *p* < 0.05 (^*^), *p* < 0.01 (**), *p* < 0.001 (^***^), and *p* < 0.0001 (^****^).

## Author Contributions

The manuscript was written with contributions from all authors. W.J., S.H.P., and S.J. conceived and designed the research. W.J. and D.Y.L. synthesized the nanoparticles and analyzed their characteristics. W.J., D.Y.L., M.A., Y.S., and Y.J. performed the in vivo experiments and conducted the US/MRI scanning. M.A., Y.J., and S.P. analyzed the MRI data. W.J., S.H.P., and S.J. analyzed the data and wrote the manuscript. All authors reviewed and approved the final version of the manuscript.

## Conflicts of Interest

S.J. is a cofounder of BiliX and ToolBio. None of these activities is related to this paper. The other authors declare that they have no competing interests.

## Supporting information




**Supporting File**: advs75692‐sup‐0001‐SuppMat.docx.

## Data Availability

The data that support the findings of this study are available from the corresponding author upon reasonable request.
