# Supplementary material for: ROS‐Responsive Nanobubbles for Dual‐Enhanced Ultrasound and Magnetic Resonance Imaging of Tumor Oxidative Stress
Source: Adv Sci (Weinh). 2026 May 13;13(44):e00071. doi: 10.1002/advs.202600071 (PMC13335788; doi:10.1002/advs.202600071)
Supplement: Supplementary file 1 [file ADVS-13-e00071-s001.docx]

**ROS-responsive nanobubbles for dual-enhanced ultrasound and magnetic resonance imaging of tumor oxidative stress**

*Wonsik Jung, Youngju Son, Dong Yun Lee, Youngeun Jeon, Muhammad Asaddudin, Sung-Hong Park* and Sangyong Jon^*^*

Dr. W. Jung, Y. Son, Prof. Dr. S. Jon

Department of Biological Sciences

KAIST Institute for BioCentury

Korea Advanced Institute of Science and Technology (KAIST)

291 Daehak-ro, Daejeon 34141, Republic of Korea
E-mail: [syjon@kaist.ac.kr](mailto:syjon@kaist.ac.kr)

Dr. W. Jung, Y. Son, Prof. Dr. S. Jon

Center for Precision Bio-Nanomedicine

Korea Advanced Institute of Science and Technology (KAIST)

291 Daehak-ro, Daejeon 34141, Republic of Korea

Prof. Dr. D. Y. Lee

Department of Nuclear Medicine

Asan Medical Center

University of Ulsan College of Medicine

88 Olympic-ro 43-gil, Seoul 05505, Republic of Korea

Y. Jeon, Dr. M. Asaduddin, Prof. Dr. S. Park

Department of Bio and Brain Engineering

Korea Advanced Institute of Science and Technology (KAIST)

291 Daehak-ro, Daejeon 34141, Republic of Korea

E-mail: [sunghongpark@kaist.ac.kr](mailto:syjon@kaist.ac.kr)

**Supplementary Figure 1. Time-dependent changes in the hydrodynamic size of bt-PEG-BR@PFP nanobubbles.** Dynamic light scattering (DLS) measurements of bt-PEG-BR@PFP nanobubbles suspended in PBS were performed at 0 h (**a**), 3 h (**b**), and 24 h (**c**) to evaluate their colloidal stability over time.

**Supplementary Figure 2. UV–vis absorbance spectra of bt-BRNPs and bt-PEG-BR@PFP nanobubbles.** The UV–vis absorbance spectrum of bt-PEG-BR@PFP is clearly distinguishable from that of bt-BRNPs, while preserving the characteristic absorption peak of bilirubin around 450 nm.

**Supplementary Figure 3. ROS-triggered size change of bt-PEG-BR@PFP nanobubbles and SonoVue® microbubbles.** **a,** Hydrodynamic size distributions of bt-PEG-BR@PFP nanobubbles before (green) and after treatment with NaOCl (150 μM, 10 min; red), as measured by dynamic light scattering (DLS). **b,** Representative photographic image showing visual changes in bt-PEG-BR@PFP nanobubbles following reaction with NaOCl (150 μM, 10 min), indicating ROS-responsive fusion and size enlargement. **c,** Dynamic light scattering measurements of SonoVue® microbubbles before NaOCl exposure (150 μM, 10 min). **d,** Dynamic light scattering measurements of SonoVue® microbubbles after NaOCl exposure (150 μM, 10 min).

**Supplementary Figure 4. ROS-mediated oxidation of bt-PEG-BR@PFP nanobubbles.** **a,** UV-vis absorption spectra of bt-PEG-BR@PFP nanobubbles ([PFP] = 71 mM; 5 mol% biotinylation) before and after exposure to NaOCl (600 μM, 10 min). **b,** Digital image of bt-PEG-BR@PFP nanobubble suspensions ([PFP] = 141.5 mM; 5 mol% biotinylation) before and after NaOCl (600 μM, 10 min) exposure.

**Supplementary Figure 5. Cell-targeting ability of bt-PEG-BR@PFP nanobubbles.** A549 cancer cells were incubated with either PEG-BR@PFP nanobubbles or bt-PEG-BR@PFP nanobubbles ([PFP] = 100 μM; 5 mol% biotinylation) for 1, 2, or 4 hours, and cellular uptake was visualized using laser-scanning confocal microscopy. For competition assays, cells were pretreated with excess free biotin (2 mM) for 1 hour prior to nanobubble incubation. Merged images show bright-field and fluorescence signals (blue: Hoechst 33342). Scale bar, 50 μm.

**Supplementary Figure 6. *In vitro* ROS responsiveness of bt-PEG-BR@PFP nanobubbles.**
bt-PEG-BR@PFP nanobubbles ([PFP] = 9.69 mM) were incubated with varying concentrations of NaOCl or H₂O₂, followed by ultrasound (US) imaging to evaluate ROS-triggered echogenicity.

**Supplementary Figure 7. Detection of ROS in A549 and DU145 tumor tissues using DHE staining.** **a,** Representative fluorescence images of tumor sections stained with dihydroethidium (DHE), acquired by laser-scanning confocal microscopy. Scale bar, 50 μm. **b, c,** Quantitative analysis of DHE-stained area (**b**) and DHE fluorescence intensity (**c**) in A549 and DU145 tumor tissues. Data are presented as mean ± S.D. (n = 3). *****P*** < 0.01, *******P*** < 0.0001 (two-tailed unpaired t-test).

**Supplementary Figure 8.** **T2 and T2* mapping of bt-PEG-BR@PFP nanobubbles in the absence or presence of NaOCl (150 μM) at increasing concentrations of PFP: (1) 6.6 mM, (2) 13.3 mM, (3) 26.5 mM, (4) 53 mM, (5) 106 mM, and (6) 212 mM.**

**Supplementary Figure 9.** **Quantitative susceptibility map (QSM) images of bt-PEG-BR@PFP nanobubbles in the absence or presence of NaOCl (150 μM) at increasing concentrations of PFP: (1) 6.6 mM, (2) 13.3 mM, (3) 26.5 mM, (4) 53 mM, (5) 106 mM, and (6) 212 mM.**

**Supplementary Figure 10.** **In vitro T2*-weighted MR phantom images of bt-PEG-BR@PFP nanobubbles ([PFP] = 9.69 mM) after 7 h incubation with increasing NaOCl concentrations.** Red arrows denote coalesced PFP-derived bubbles.

**Supplementary Figure 11.** **Active tumor-targeting ability of cypate-loaded bt-PEG-BR@PFP nanobubbles in A549 human lung tumor-bearing mice. a,** Schematic illustration of the preparation of cypate-loaded bt-PEG-BR@PFP nanobubbles for *in vivo* fluorescence tracking. **b,** *In vivo* fluorescence images of A549 tumor-bearing mice at different time points after intravenous (retro-orbital) injection of cypate-loaded bt-PEG-BR@PFP nanobubbles (1.4 mg cypate/kg). Yellow dotted lines delineate tumor boundaries. The color bar represents the relative fluorescence radiance. **c,** *Ex vivo* IVIS fluorescence images of major organs collected 6 h post-injection. **d,** *Ex vivo* fluorescence images of excised tumors collected 6 h post-injection from mice treated with free cypate or cypate-loaded bt-PEG-BR@PFP nanobubbles (1.4 mg cypate/kg). **e,** Quantitative analysis of tumor fluorescence intensity based on *ex vivo* imaging data. Data are shown as mean ± s.e.m (n = 3, *P < 0.05; two-tailed unpaired t-test).

**Supplementary Figure 12.** **Pharmacokinetic profiles of cypate-loaded bt-PEG-BR@PFP nanobubbles.** Blood concentration-time profile of cypate after intravenous (r.o.) injection of cypate-loaded bt-PEG-BR@PFP nanobubbles. Blood samples were collected at 0 min, 5 min, 30 min, 1 h, 2 h, 4 h, 8 h, 12 h, 24 h, and 48 h post-injection. Data were fitted to a two-compartment model to determine pharmacokinetic parameters using PKSolver software (n = 3).

**Supplementary Figure 13.** ***In vivo* safety evaluation of bt-PEG-BR@PFP nanobubbles ([PFP] = 100 mg/kg). a,** Changes in body weight of mice. PBS or bt-PEG-BR@PFP was injected intravenously (r.o.) on day 0 and body weights of mice were monitored for 7 days. Data are presented as mean ± s.e.m (n = 3, n.s., not significant; two-way ANOVA). **b,** Blood test parameters for mice treated with PBS or bt-PEG-BR@PFP nanobubbles via a single intravenous (r.o.) injection on day 0. Blood sample were collected 7 days after intravenous (r.o.) injection. Data are presented as mean ± s.e.m (n = 3, *P < 0.05; two-tailed unpaired t-test). **c,** Histopathological analysis of major organs. The heart, lung, liver, spleen and kidney tissues from PBS or bt-PEG-BR@PFP nanobubbles intravenously (r.o.) injected mice groups were stained with H&E. Scale bar, 50 μm.
